# Early Onset Active Inflammatory Bowel Disease Is Associated With Psychiatric Comorbidities: A Multi-Network Propensity-Matched Cohort Study

**DOI:** 10.1093/crocol/otae066

**Published:** 2024-12-20

**Authors:** Ahmed Nadeem, Sydney Donohue, Fatima Zehra Shah, Jaime Abraham Perez, Elleson Harper, Preetika Sinh, Ruthvik Padival, Gregory Cooper, Jeffry Katz, Fabio Cominelli, Miguel Regueiro, Emad Mansoor

**Affiliations:** Department of Internal Medicine, Cleveland Clinic Foundation, Cleveland, OH, USA; Department of Internal Medicine, Case Western Reserve University/University Hospitals Cleveland Medical Center, Cleveland, OH, USA; Department of Pediatrics, Cleveland Clinic Foundation, Cleveland, OH, USA; Clinical Research Center, University Hospitals Cleveland Medical Center, Cleveland, OH, USA; Clinical Research Center, University Hospitals Cleveland Medical Center, Cleveland, OH, USA; Division of Gastroenterology and Hepatology, Medical College of Wisconsin, Milwaukee, WI, USA; Intermountain Gastroenterology Specialists, Murray, UT, USA; Digestive Health Institute, Case Western Reserve University/University Hospitals Cleveland Medical Center, Cleveland, OH, USA; Digestive Health Institute, Case Western Reserve University/University Hospitals Cleveland Medical Center, Cleveland, OH, USA; Digestive Health Institute, Case Western Reserve University/University Hospitals Cleveland Medical Center, Cleveland, OH, USA; Department of Gastroenterology, Hepatology, and Nutrition, Digestive Disease and Surgery Institute, Cleveland Clinic Foundation, Cleveland, OH, USA; Digestive Health Institute, Case Western Reserve University/University Hospitals Cleveland Medical Center, Cleveland, OH, USA

**Keywords:** inflammatory bowel disease, psychiatric diagnoses, psychotropic medications, ulcerative colitis, Crohn’s disease

## Abstract

**Background:**

Psychiatric disease burden in patients with Inflammatory bowel disease (IBD) has risen substantially over the past few decades. However, there is limited data on the relationship between IBD disease activity and the incidence of psychiatric comorbidities. We sought to conduct a population-based study to investigate the impact of early onset disease activity in newly diagnosed IBD patients on psychiatric disease diagnoses and medication usage.

**Methods:**

We performed a retrospective cohort study using the TriNetX database. We identified all adult patients diagnosed with IBD and documented IBD-specific medication use. We stratified these IBD patients into 2 cohorts based on IBD Disease Activity, occurring 6 months to 1 year after initial IBD diagnosis. Active IBD was defined as the utilization of steroids and/or elevated fecal calprotectin [≥200 µg/g] occurring 6 months to 1 year after initial IBD diagnosis. We examined the outcomes of psychiatric disease diagnoses and psychotropic medication prescriptions occurring 1 year after the initial diagnosis.

**Results:**

Out of 69 105 patients with an IBD diagnosis during the study period, after propensity score matching, 16 922 IBD patients each were included in the 2 cohorts based on disease activity. Patients with active IBD had significantly higher odds of developing major depressive disorder, anxiety disorder, bipolar disorder, alcohol use disorder, opiate use disorder, attention deficit hyperactivity disorder, and obsessive-compulsive disorder. Additionally, patients with active IBD also had significantly higher odds of using all studied psychotropic medications, including antidepressants, antipsychotic medications, anxiolytics, sedatives, hypnotic medications, mood stabilizers, stimulant medications, and medications used for substance use disorders (including alcohol, opioid, and tobacco use).

**Conclusions:**

Active IBD shortly after the IBD diagnosis is associated with a higher incidence of psychiatric comorbidities. Awareness of behavioral health in IBD is important, and proper treatment is necessary.

## Graphical Abstract

**Graphical Abstract:**
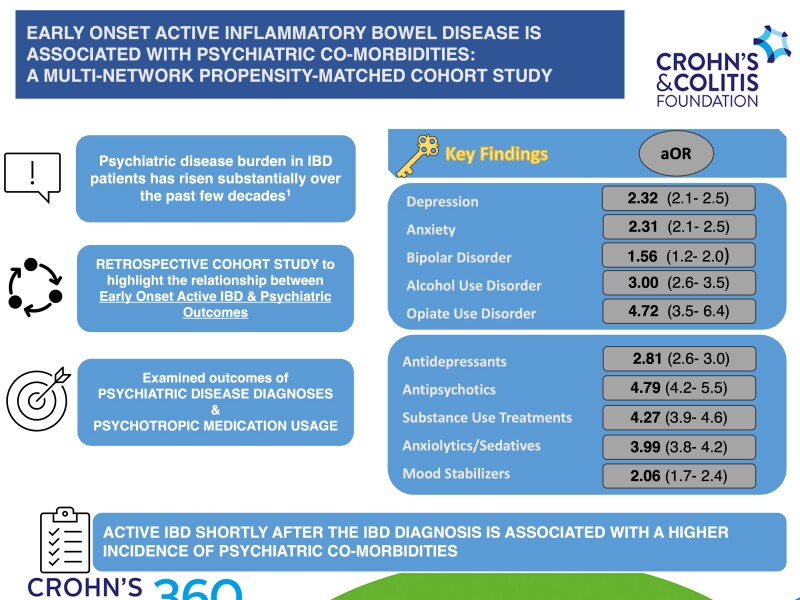

## Introduction

Inflammatory bowel disease (IBD) is a chronic remitting and relapsing condition with a high socioeconomic burden. Inflammatory bowel disease patients have a higher prevalence of comorbid conditions, with concurrent psychiatric disease diagnoses accounting for a substantial part of their disability, especially in patients with poorly controlled disease.^1^ Prior studies have shown that IBD patients with comorbid psychiatric disease have a substantially higher utilization of inpatient, emergency, and surgical services as compared to those patients without a concurrent psychiatric comorbid condition.^2^ Multiple studies have demonstrated a bi-directional relationship between psychiatric comorbid conditions and IBD, where the presence of psychiatric disease can influence IBD disease course and utilization of therapeutics.^3^ In our study, we sought to evaluate the impact of early onset IBD Disease Activity on concurrent psychiatric diagnoses and psychotropic medication prescription use. We hypothesize that patients with active IBD early in their disease course experience increased psychiatric disease severity, comorbid psychiatric diagnoses, and increased psychotropic and nonpsychotropic medication usage.

## Methods

### Data Source and Selection

We performed a retrospective cohort study using a large de-identified national database with 80 contributing healthcare organizations (HCO), including over 113 million patients (TriNetX Research Network, Cambridge, MA; date of access: November 16, 2023; IRB exempt). We identified all adult patients (≥18 years of age) newly diagnosed with IBD (Crohn’s disease (CD) and ulcerative colitis (UC)) (ICD-10: K50, K51) and documented IBD-specific medication use (adalimumab, certolizumab pegol, golimumab, infliximab, vedolizumab, ustekinumab, tofacitinib, azathioprine, mercaptopurine, or methotrexate), on or after January 1, 2013. This was done to ensure a higher positive predictive value for IBD diagnosis.^4^

We stratified these IBD patients into 2 cohorts based on IBD Disease Activity, occurring 6 months to 1 year after initial IBD diagnosis. Active IBD was defined as the utilization of steroids (hydrocortisone, prednisone, methylprednisolone, or budesonide) and/or elevated fecal calprotectin (FC) [≥200 µg/g] occurring 6 months to 1 year after initial IBD diagnosis. We did not define relative disease activity in our study since the prescription of steroids and elevated FC > 200 at 6-12 months would represent active IBD regardless of disease activity at diagnosis or between 0 and 6 months. This could represent a new flare or persistent disease activity since diagnosis. Nonactive IBD was defined as nonutilization of steroids and without elevated FC occurring 6 months to 1 year after initial IBD diagnosis.

We excluded all patients with prior diagnoses of psychological disorders, including major depressive disorder (MDD), anxiety disorder, bipolar disorder, alcohol and opiate use disorder, attention deficit hyperactivity disorder (ADHD), and obsessive-compulsive disorder (OCD) (based on respective ICD-10 codes as mentioned in Supplementary Table 1A). We also excluded prior psychotropic medication use based on RxNorm codes, including antidepressants, antipsychotics, medications used for substance use disorders, anxiolytics/sedatives or hypnotic medications, mood stabilizers, stimulants, or other psychiatric-related medications (all specific medications excluded are in Supplementary Table 1B). We examined the outcomes of psychiatric disease diagnoses (including MDD, anxiety disorder, bipolar disorder, alcohol and opiate use disorder, ADHD, and OCD) based on ICD-10 codes and psychotropic medication prescriptions (based on medications listed above according to RxNorm codes listed in Supplementary Table 1B) occurring at least 1 year after the initial IBD diagnosis.

Additionally, we performed a sensitivity analysis and stratified IBD patients into UC and CD patients. Between each disease class, we also examined the outcomes of psychiatric disease diagnoses and psychotropic medication prescriptions occurring at least 1 year after initial UC or CD diagnosis between patients with active UC or CD. We compared it with those without active IBD, respectively. All ICD-10 codes used for diagnoses are listed in Supplementary Table 1A.

### Statistical Analysis

We performed 1:1 greedy nearest neighbor propensity score matching (caliper of 0.1 standard deviations) to balance cohorts on age, sex, race, ethnicity, comorbid conditions of the Charlson Comorbidity Index (including cardiovascular, pulmonary, endocrine, rheumatologic, renal comorbidities, neoplasms, anal disease, etcetera), procedure history (including colectomy and other surgical procedures of the colon, rectum, intestines, and stomach) as well emergency department visits (Table 1). We calculated baseline differences between groups using an independent *t*-test (continuous data), chi-square test, or Fisher’s exact test (categorical data) as appropriate. Estimates of odds ratios (OR), and 95% confidence intervals (CI) were used to describe the outcome risks. We included the standardized mean difference (SMD) in our propensity-matched tables. The majority of the variables resulted in an SMD < 0.1, which indicates sufficient balance. For the few variables that resulted in SMD > 0.1, we believe this is a result of the rounding procedure implemented by TriNetX. TriNetX automatically rounds patient counts of 1-9, up to 10, which may affect associations with small patient counts. As a result, analyses with small patient counts can provide misleading information. *P*-values of *α* < .05 were considered statistically significant.

**Table 1.** Baseline characteristics for patients with active IBD and without active IBD, before and after propensity score matching.

	Before matching				After matching			
	Active IBD	No active IBD	*P*-value	SMD	Active IBD	No active IBD	*P*-value	SMD
*n*	16 961	52 144			16 922	16 922		
Age at Index	43.7 ± 17.56	44.46 ± 18.48	**<.001**	0.042	43.69 ± 17.55	43.38 ± 17.83	.108	0.017
Sex								
Male	7586 (44.7%)	25 264 (48.5%)	**<.001**	0.075	7578 (44.8%)	7694 (45.5%)	.205	0.014
Female	8199 (48.3%)	25 282 (48.5%)	.743	0.003	8190 (48.4%)	8094 (47.8%)	.296	0.011
Unknown Gender	1176 (6.9%)	1598 (3.1%)	**<.001**	0.178	1154 (6.8%)	1134 (6.7%)	.665	0.005
Race								
White	11 891 (70.1%)	37 562 (72.0%)	**<.001**	0.043	11 883 (70.2%)	11 868 (70.1%)	.859	0.002
Black or African American	1453 (8.6%)	3180 (6.1%)	**<.001**	0.095	1448 (8.6%)	1530 (9.0%)	.116	0.017
Asian	421 (2.5%)	1300 (2.5%)	.937	0.001	421 (2.5%)	385 (2.3%)	.199	0.014
American Indian or Alaska Native	37 (0.2%)	126 (0.2%)	.584	0.005	37 (0.2%)	38 (0.2%)	.908	0.001
Native Hawaiian or Other Pacific Islander	27 (0.2%)	27 (0.1%)	**<.001**	0.033	23 (0.1%)	21 (0.1%)	.763	0.003
Other Race	549 (3.2%)	2012 (3.9%)	**<.001**	0.034	549 (3.2%)	526 (3.1%)	.476	0.008
Unknown Race	2583 (15.2%)	7937 (15.2%)	.981	0.000	2561 (15.1%)	2554 (15.1%)	.915	0.001
Ethnicity								
Hispanic or Latino	793 (4.7%)	2235 (4.3%)	**.031**	0.019	791 (4.7%)	755 (4.5%)	.349	0.010
Not Hispanic or Latino	12 207 (72.0%)	34 404 (66.0%)	**<.001**	0.130	12 192 (72.0%)	12 213 (72.2%)	.799	0.003
Unknown Ethnicity	3961 (23.4%)	15 505 (29.7%)	**<.001**	0.145	3939 (23.3%)	3954 (23.4%)	.847	0.002
Comorbidities								
Acute myocardial infarction	107 (0.6%)	514 (1.0%)	**<.001**	0.040	107 (0.6%)	79 (0.5%)	**.040**	0.022
Anal abscess	149 (0.9%)	547 (1.0%)	.053	0.017	148 (0.9%)	131 (0.8%)	.307	0.011
Anal fistula	251 (1.5%)	915 (1.8%)	**.016**	0.022	249 (1.5%)	223 (1.3%)	.228	0.013
Chronic kidney disease (CKD)	530 (3.1%)	1461 (2.8%)	**.029**	0.019	525 (3.1%)	452 (2.7%)	**.018**	0.026
Chronic vascular disorders of intestine	13 (0.1%)	49 (0.1%)	.513	0.006	13 (0.1%)	14 (0.1%)	.847	0.002
Diabetes mellitus	959 (5.7%)	3230 (6.2%)	**.010**	0.023	954 (5.6%)	922 (5.4%)	.447	0.008
Diseases of liver	814 (4.8%)	2492 (4.8%)	.915	0.001	813 (4.8%)	760 (4.5%)	.171	0.015
Fistula of intestine	206 (1.2%)	436 (0.8%)	**<.001**	0.038	200 (1.2%)	180 (1.1%)	.302	0.011
Heart failure	241 (1.4%)	938 (1.8%)	**.001**	0.030	238 (1.4%)	208 (1.2%)	.153	0.016
Human immunodeficiency virus (HIV) disease	64 (0.4%)	218 (0.4%)	.470	0.006	64 (0.4%)	51 (0.3%)	.225	0.013
Ischiorectal abscess	81 (0.5%)	343 (0.7%)	**.009**	0.024	81 (0.5%)	74 (0.4%)	.573	0.006
Neoplasms	2447 (14.4%)	8288 (15.9%)	**<.001**	0.041	2436 (14.4%)	2361 (14.0%)	.242	0.013
Nicotine dependence	917 (5.4%)	2319 (4.4%)	**<.001**	0.044	906 (5.4%)	862 (5.1%)	.282	0.012
Other and unspecified intestinal obstruction	607 (3.6%)	1306 (2.5%)	**<.001**	0.063	594 (3.5%)	549 (3.2%)	.176	0.015
Other cerebrovascular diseases	87 (0.5%)	430 (0.8%)	**<.001**	0.038	87 (0.5%)	79 (0.5%)	.534	0.007
Other chronic obstructive pulmonary disease	403 (2.4%)	774 (1.5%)	**<.001**	0.065	388 (2.3%)	382 (2.3%)	.827	0.002
Other peripheral vascular diseases	191 (1.1%)	683 (1.3%)	.063	0.017	191 (1.1%)	164 (1.0%)	.150	0.016
Other rheumatoid arthritis	255 (1.5%)	842 (1.6%)	.314	0.009	255 (1.5%)	228 (1.3%)	.216	0.013
Overweight and obesity	978 (5.8%)	3420 (6.6%)	**<.001**	0.033	975 (5.8%)	934 (5.5%)	.334	0.011
Peptic ulcer, site unspecified	85 (0.5%)	313 (0.6%)	.138	0.013	82 (0.5%)	70 (0.4%)	.329	0.011
Rectal abscess	158 (0.9%)	555 (1.1%)	.137	0.013	157 (0.9%)	139 (0.8%)	.293	0.011
Rheumatoid arthritis with rheumatoid factor	28 (0.2%)	145 (0.3%)	**.011**	0.024	28 (0.2%)	22 (0.1%)	.396	0.009
Systemic connective tissue disorders	305 (1.8%)	838 (1.6%)	.090	0.015	303 (1.8%)	286 (1.7%)	.480	0.008
Unspecified dementia	23 (0.1%)	231 (0.4%)	**<.001**	0.057	23 (0.1%)	19 (0.1%)	.537	0.007
Unspecified intestinal obstruction	458 (2.7%)	951 (1.8%)	**<.001**	0.059	448 (2.6%)	408 (2.4%)	.166	0.015
Procedures								
Colectomy, partial	29 (0.2%)	86 (0.2%)	.867	0.001	29 (0.2%)	25 (0.1%)	.586	0.006
Surgical procedures on the colon and rectum	2305 (13.6%)	11 447 (22.0%)	**<.001**	0.220	2303 (13.6%)	2237 (13.2%)	.292	0.011
Surgical procedures on the intestines (except rectum)	385 (2.3%)	1239 (2.4%)	.428	0.007	382 (2.3%)	366 (2.2%)	.554	0.006
Surgical procedures on the stomach	14 (0.1%)	100 (0.2%)	**.002**	0.030	14 (0.1%)	14 (0.1%)	1.000	0.000
Visit: Emergency	3171 (18.7%)	8777 (16.8%)	**<.001**	0.049	3149 (18.6%)	3025 (17.9%)	.081	0.019
Emergency Department Services	2698 (15.9%)	7262 (13.9%)	**<.001**	0.056	2672 (15.8%)	2543 (15.0%)	.052	0.021

Significant *P*-values in bold.

For the query on missing data, a few patients did not report race/ethnicity data. However, this did not affect our propensity-matched approach, as numerous other variables were included in the matching criteria, resulting in a similar proportion of unknown race/ethnicity patients. Importantly, due to the structure of our queries, these patients include follow-up data, which may indicate that the unknown race/ethnicity data represents a “patient declined” variable rather than a true missing variable, which is much more common in patients with a single HCO interaction. No other variables were identified as missing or requiring some imputation, resulting in an average of 2880 patient facts per individual.

### Secondary Analysis

As a secondary analysis to mitigate the potential confounding effect of steroids on psychiatric diagnoses, we also performed a separate sensitivity analysis by using elevated FC [≥ 200 µg/g] alone and using steroid medications alone to define active IBD and compare it to patients with nonactive IBD.

## Results

### Baseline Characteristics

We identified 69 105 patients who carried a diagnosis of IBD during the study period between 2013 and 2023. Of these, 16 961 (24.5%) had an IBD exacerbation occurring 6 months to 1 year after their initial diagnosis. After propensity score matching, we identified 16 922 IBD patients who had disease exacerbation and 16 922 IBD patients who did not have any disease exacerbation. The mean age of the active IBD cohort was 43.69 ± 17.55; 48.4% were female, and 70.2% were white. Propensity score matching was successful for all measured covariates. A complete list of all demographic parameters, comorbid conditions, and surgical procedures can be found in Table 1.

### Outcomes

#### All IBD patients

##### Psychiatric disease diagnoses.

For the outcome of psychiatric disease diagnosis, patients with active IBD (occurring 6 months to 1 year after their initial diagnosis) compared with patients without active IBD had significantly higher odds of having a subsequent psychiatric diagnosis across all specific diseases, as shown in Table 2A Specifically, patients with active IBD had higher odds of developing MDD [12.2% vs. 5.7% aOR: 2.32 95% CI, 2.14-2.51] and anxiety disorder [15.3% vs. 7.2% aOR: 2.31 95% CI, 2.15-2.48]. Patients with increased IBD activity were also more likely to develop bipolar disorder [1.0% vs. 0.7% aOR: 1.56 95% CI, 1.23-1.99], alcohol use disorder [3.5% vs. 1.2% aOR: 3.0 95% CI, 2.55-3.53], opiate use disorder [1.4% vs. 0.3% aOR: 4.72 95% CI, 3.48-6.39], ADHD [1.3% vs. 1.0% aOR: 1.34 95% CI, 1.09-1.64] and obsessive-compulsive disorder [0.4% vs. 0.2% aOR: 2.0 95% CI, 1.36-2.96].

**Table 2.** (A) and (B) Psychiatric disease outcomes and psychotropic medication prescription between patients who have active who did vs. those who did not, after propensity score matching, with OR and 95% CI.

Psychiatric disease	Active IBD	No active IBD	OR (95% CI)	*P*-value
A. Psychiatric disease diagnosis				
Depression	2067 (12.2%)	957 (5.7%)	2.32 (2.14, 2.51)	**<.001**
Anxiety	2585 (15.3%)	1226 (7.2%)	2.31 (2.15, 2.48)	**<.001**
Bipolar disorder	173 (1.0%)	111 (0.7%)	1.56 (1.23, 1.99)	**<.001**
Alcohol use disorder	589 (3.5%)	201 (1.2%)	3.00 (2.55, 3.53)	**<.001**
Opiate use disorder	238 (1.4%)	51 (0.3%)	4.72 (3.48, 6.39)	**<.001**
ADHD	221 (1.3%)	166 (1.0%)	1.34 (1.09, 1.64)	**.005**
OCD	76 (0.4%)	38 (0.2%)	2.00 (1.36, 2.96)	**<.001**
Medications	Active IBD	No active IBD	OR (95% CI)	*P*-value
B. Psychotropic medication prescription				
Antidepressants	3861 (22.8%)	1611 (9.5%)	2.81 (2.64, 2.99)	**<.001**
Antipsychotics	1267 (7.5%)	281 (1.7%)	4.79 (4.2, 5.46)	**<.001**
Substance use treatments	2793 (16.5%)	749 (4.4%)	4.27 (3.93, 4.64)	**<.001**
Anxiolytics/sedatives/hypnotics	5928 (35.0%)	2013 (11.9%)	3.99 (3.78, 4.22)	**<.001**
Mood stabilizers	415 (2.5%)	204 (1.2%)	2.06 (1.74, 2.44)	**<.001**
Stimulants	276 (1.6%)	207 (1.2%)	1.34 (1.12, 1.61)	**.002**

Significant OR and *P*-values in bold.

##### Psychotropic medication use.

For the outcome of psychotropic medication usage, patients with active IBD (occurring 6 months to 1 year after their initial diagnosis) compared with patients without active IBD had significantly higher odds of using all studied psychotropic medications, as shown in Table 2B. Specifically, patients with active IBD had higher utilization of antidepressants [22.8% vs. 9.5% aOR: 2.81 95% CI, 2.64-2.99], antipsychotic medications [7.5% vs. 1.7% aOR: 4.79 95% CI, 4.20-5.46] and anxiolytics/sedatives/hypnotic medication usage [35.0% vs. 11.9% aOR: 3.99 95% CI, 3.78-4.22]. Patients with active IBD were also more likely to have been prescribed mood stabilizers [2.5% vs. 1.2% aOR: 2.06 95% CI, 1.74-2.44] and stimulant medications [1.6% vs. 1.2% aOR: 1.34 95% CI, 1.12-1.61]. Medications used for substance use disorders (including alcohol, opioid, and tobacco use) were also prescribed more for patients with active IBD [16.5% vs. 4.4% aOR: 4.27 95% CI, 3.93-4.64] as compared to those without.

### Sensitivity Analyses

#### CD patients

##### Psychiatric disease diagnoses.

For the outcome of psychiatric disease diagnosis, patients with active CD (occurring 6 months to 1 year after their initial diagnosis) compared with patients without active IBD had significantly higher odds of having a subsequent psychiatric diagnosis across all specific diseases, as shown in Table 3A. Specifically, patients with active CD had higher odds of developing MDD [15.3% vs. 5.7% aOR: 3.02 95% CI, 2.74-3.34] and anxiety disorder [18.3% vs. 7.6% aOR: 2.7 95% CI, 2.48-2.95]. Patients with increased CD activity were also more likely to develop Bipolar Disorder [1.7% vs. 0.7% aOR: 2.54 95% CI, 1.92-3.37], alcohol use disorder [4.2% vs. 1.2% aOR: 3.57 95% CI, 2.92-4.36], opiate use disorder [2.1% vs. 0.4% aOR: 5.31 95% CI, 3.81-7.39], ADHD [1.6% vs. 0.9% aOR: 1.78 95% CI, 1.39-2.29] and obsessive-compulsive disorder [0.6% vs. 0.2% aOR: 2.83 95% CI, 1.74-4.6].

**Table 3.** (A) and (B) Psychiatric disease outcomes and psychotropic medication prescription between patients with active CD and without active IBD, after propensity score matching, with OR and 95% CI.

Psychiatric disease	Active CD	Inactive IBD	OR (95% CI)	*P*-value
A. Psychiatric disease diagnosis				
Depression	1590 (15.3%)	586 (5.7%)	3.02 (2.74, 3.34)	**<.001**
Anxiety	1892 (18.3%)	791 (7.6%)	2.7 (2.48, 2.95)	**<.001**
Bipolar disorder	171 (1.7%)	68 (0.7%)	2.54 (1.92, 3.37)	**<.001**
Alcohol use disorder	436 (4.2%)	126 (1.2%)	3.57 (2.92, 4.36)	**<.001**
Opiate use disorder	219 (2.1%)	42 (0.4%)	5.31 (3.81, 7.39)	**<.001**
ADHD	170 (1.6%)	96 (0.9%)	1.78 (1.39, 2.29)	**<.001**
OCD	62 (0.6%)	22 (0.2%)	2.83 (1.74, 4.6)	**<.001**
Medications	Active CD	Inactive IBD	OR (95% CI)	*P*-value
B. Psychotropic medication prescription				
Antidepressants	2781 (26.8%)	1046 (10.1%)	3.27 (3.02, 3.53)	**<.001**
Antipsychotics	951 (9.2%)	176 (1.7%)	5.85 (4.97, 6.89)	**<.001**
Substance use treatments	2055 (19.8%)	445 (4.3%)	5.51 (4.96, 6.13)	**<.001**
Anxiolytics/sedatives/hypnotics	4117 (39.7%)	1276 (12.3%)	4.69 (4.37, 5.04)	**<.001**
Mood stabilizers	334 (3.2%)	140 (1.4%)	2.43 (1.99, 2.97)	**<.001**
Stimulants	202 (1.9%)	123 (1.2%)	1.66 (1.32, 2.07)	**<.001**

Significant OR and *P*-values in bold.

##### Psychotropic medication use.

For the outcome of psychotropic medication usage, patients with active CD (occurring 6 months to 1 year after their initial diagnosis) compared with patients without active IBD had significantly higher odds of using all studied psychotropic medications, as shown in Table 3B. Specifically, patients with active CD had higher utilization of antidepressants [26.8% vs. 10.1% aOR: 3.27 95% CI, 3.02-3.53], antipsychotic medications [9.2% vs. 1.7% aOR: 5.85 95% CI, 4.97-6.89] and anxiolytics/sedatives/hypnotic medication usage [39.7% vs. 12.3% aOR: 4.69 95% CI, 4.37-5.04]. Patients with active CD were also more likely to have been prescribed mood stabilizers [3.2% vs. 1.4% aOR: 2.43 95% CI, 1.99-2.97] and stimulant medications [1.9% vs. 1.2% aOR: 1.66 95% CI, 1.32-2.07]. Medications used for substance use disorders (including alcohol, opioid, and tobacco use) were also prescribed more for patients with active CD [19.8% vs. 4.3% aOR: 5.51 95% CI, 4.96-6.13] as compared to those without active IBD.

#### UC patients

##### Psychiatric disease diagnoses.

For the outcome of psychiatric disease diagnosis, patients with active UC (occurring 6 months to 1 year after their initial diagnosis) compared with patients without active IBD had significantly higher odds of having a subsequent psychiatric diagnosis across most of the specific psychiatric diseases (except Bipolar disorder), as shown in Table 4A. Specifically, patients with active UC had higher odds of developing MDD [12.5% vs. 5.3% aOR: 2.59 95% CI, 2.32-2.89] and anxiety disorder [15.9% vs. 6.8% aOR: 2.6 95% CI, 2.35-2.86]. However, patients with increased UC activity were not more likely to develop bipolar disorder [0.6% vs. 0.6% aOR: 0.9 95% CI, 0.62-1.3]. Additionally, patients with UC had higher odds to develop alcohol use disorder [3.8% vs. 1.0% aOR: 3.79 95% CI, 3.02-4.76], opiate use disorder [1.1% vs. 0.3% aOR: 3.15 95% CI, 2.11-4.69], ADHD [1.3% vs. 0.9% aOR: 1.54 95% CI, 1.16-2.04] and OCD [0.4% vs. 0.2% aOR: 1.73 95% CI, 1.02-2.93].

**Table 4.** (A) and (B) Psychiatric disease outcomes and psychotropic medication prescription between patients with active UC and without active IBD, after propensity score matching, with OR and 95% CI.

Psychiatric disease	Active UC	Inactive IBD	OR (95% CI)	*P*-value
A. Psychiatric disease diagnosis				
Depression	1170 (12.5%)	490 (5.3%)	2.59 (2.32, 2.89)	**<.001**
Anxiety	1481 (15.9%)	632 (6.8%)	2.6 (2.35, 2.86)	**<.001**
Bipolar disorder	54 (0.6%)	60 (0.6%)	0.9 (0.62, 1.3)	.573
Alcohol use disorder	354 (3.8%)	96 (1.0%)	3.79 (3.02, 4.76)	**<.001**
Opiate use disorder	100 (1.1%)	32 (0.3%)	3.15 (2.11, 4.69)	**<.001**
ADHD	124 (1.3%)	81 (0.9%)	1.54 (1.16, 2.04)	**.003**
OCD	38 (0.4%)	22 (0.2%)	1.73 (1.02, 2.93)	**.039**
Medications	Active UC	Inactive IBD	OR (95% CI)	*P*-value
B. Psychotropic medication prescription				
Antidepressants	2173 (23.3%)	860 (9.2%)	2.99 (2.75, 3.26)	**<.001**
Antipsychotics	678 (7.3%)	134 (1.4%)	5.38 (4.46, 6.49)	**<.001**
Substance use treatments	1617 (17.3%)	414 (4.4%)	4.51 (4.04, 5.05)	**<.001**
Anxiolytics/sedatives/hypnotics	3465 (37.1%)	1084 (11.6%)	4.49 (4.17, 4.85)	**<.001**
Mood stabilizers	217 (2.3%)	110 (1.2%)	2 (1.58, 2.52)	**<.001**
Stimulants	156 (1.7%)	104 (1.1%)	1.51 (1.18, 1.94)	**.001**

Significant OR and *P*-values in bold.

##### Psychotropic medication use.

For the outcome of psychotropic medication usage, patients with active UC (occurring 6 months to 1 year after their initial diagnosis) compared with patients without active IBD had significantly higher odds of using all studied psychotropic medications, as shown in Table 4B. Specifically, patients with active UC had higher utilization of antidepressants [23.3% vs. 9.2% aOR: 2.99 95% CI, 2.75-3.26], antipsychotic medications [7.3% vs. 1.4% aOR: 5.38 95% CI, 4.46-6.49] and anxiolytics/sedatives/hypnotic medication usage [37.1% vs. 11.6% aOR: 4.49 95% CI, 4.17-4.85]. Patients with active UC were also more likely to have been prescribed mood stabilizers [2.3% vs. 1.2% aOR: 2.0 95% CI, 1.58-2.52] and stimulant medications [1.7% vs. 1.1% aOR: 1.51 95% CI, 1.18-1.94]. Medications used for substance use disorders (including alcohol, opioid, and tobacco use) were also prescribed more for patients with active UC [17.3% vs. 4.4% aOR: 4.51 95% CI, 4.04-5.05] as compared to those without active IBD.

#### Elevated FC to define active IBD

When we used elevated FC as the sole criteria to define active IBD occurring 6-12 months after initial IBD diagnosis, we were only able to stratify 984 patients into the active IBD and the nonactive IBD cohorts, respectively (after propensity score matching). For the outcome of psychiatric disease diagnoses, IBD patients with elevated FC had significantly higher odds of developing MDD, anxiety disorder, and alcohol use disorder. Owing to the small size, we were not able to get the appropriate number of subjects to effectively highlight any statistically significant differences between the cohorts for the outcomes of bipolar disorder, ADHD, and OCD; hence, there was no significant difference between active and nonactive IBD cohorts for these outcomes. Similarly, for the outcomes of psychotropic medication usage, IBD patients with elevated FC had significantly higher odds of using antidepressants, antipsychotics, substance use treatments, and anxiolytics/sedatives/hypnotics medications. However, owing to the small size, we were not able to get the appropriate numbers to effectively highlight any statistically significant differences between the cohorts for the outcomes of mood stabilizers and stimulant medication usage. This is shown in Table 5A and B.

**Table 5.** (A) and (B) Psychiatric disease outcomes and psychotropic medication prescriptions in patients with active IBD (defined by only FC) and without active IBD, after propensity score matching, with OR and 95% CI.

Psychiatric disease	Active IBD: Calprotectin	Inactive IBD	OR (95% CI)	*P*-value
A. Psychiatric disease diagnosis				
Depression	67 (6.8%)	42 (4.3%)	1.64 (1.1, 2.44)	**.014**
Anxiety	98 (10.0%)	53 (5.4%)	1.94 (1.37, 2.75)	**<.001**
Bipolar disorder	10 (1.0%)	10 (1.0%)	1 (0.41, 2.41)	1.000
Alcohol use disorder	35 (3.6%)	10 (1.0%)	3.59 (1.77, 7.3)	**<.001**
Opiate use disorder	10 (1.0%)	10 (1.0%)	1 (0.41, 2.41)	1.000
ADHD	10 (1.0%)	10 (1.0%)	1 (0.41, 2.41)	1.000
OCD	10 (1.0%)	10 (1.0%)	1 (0.41, 2.41)	1.000
Medications	Active IBD: Calprotectin	Inactive IBD	OR (95% CI)	*P*-value
B. Psychotropic medication prescription				
Antidepressants	146 (14.8%)	76 (7.7%)	2.08 (1.55, 2.79)	**<.001**
Antipsychotics	58 (5.9%)	15 (1.5%)	4.05 (2.28, 7.19)	**<.001**
Substance use treatments	111 (11.3%)	32 (3.3%)	3.78 (2.53, 5.66)	**<.001**
Anxiolytics/sedatives/hypnotics	240 (24.4%)	104 (10.6%)	2.73 (2.13, 3.5)	**<.001**
Mood stabilizers	10 (1.0%)	10 (1.0%)	1 (0.41, 2.41)	1.000
Stimulants	10 (1.0%)	11 (1.1%)	0.91 (0.38, 2.15)	.826

Significant OR and *P*-values are in bold.

#### Steroid medication usage to define active IBD

When we used elevated steroid medication use (hydrocortisone, prednisone, methylprednisolone, or budesonide) as the sole criteria to define active IBD occurring 6-12 months after initial IBD diagnosis, we were able to stratify 16 505 patients into the active IBD and the nonactive IBD cohorts, respectively (after propensity score matching). For the outcomes of psychiatric disease diagnoses, IBD patients with steroid medication usage had significantly higher odds of developing all studied psychiatric diagnoses. Similarly, for the outcomes of psychotropic medication usage, IBD patients with steroid medication usage had significantly higher odds of using all studied psychotropic medications. This is shown in Table 6A and B.

**Table 6.** (A) and (B) Psychiatric disease outcomes and psychotropic medication prescription between patients with active IBD (defined by only glucocorticoid usage) and without active IBD, after propensity score matching, with OR and 95% CI.

Psychiatric Disease	Active IBD: Glucocorticoids	Inactive IBD	OR (95% CI)	*P*-value
A. Psychiatric disease diagnosis				
Depression	2041 (12.4%)	942 (5.7%)	2.33 (2.15, 2.53)	**<.001**
Anxiety	2544 (15.4%)	1218 (7.4%)	2.29 (2.13, 2.46)	**<.001**
Bipolar disorder	173 (1.0%)	109 (0.7%)	1.59 (1.25, 2.03)	**<.001**
Alcohol use disorder	576 (3.5%)	211 (1.3%)	2.79 (2.38, 3.27)	**<.001**
Opiate use disorder	237 (1.4%)	54 (0.3%)	4.44 (3.3, 5.97)	**<.001**
ADHD	221 (1.3%)	159 (1.0%)	1.4 (1.14, 1.71)	**.001**
OCD	75 (0.5%)	40 (0.2%)	1.88 (1.28, 2.76)	**.001**
Medications	Active IBD: Glucocorticoids	Inactive IBD	OR (95% CI)	*P*-value
B. Psychotropic medication prescription				
Antidepressants	3815 (23.1%)	1617 (9.8%)	2.77 (2.6, 2.95)	**<.001**
Antipsychotics	1245 (7.5%)	282 (1.7%)	4.69 (4.12, 5.35)	**<.001**
Substance use treatments	2763 (16.7%)	723 (4.4%)	4.39 (4.03, 4.78)	**<.001**
Anxiolytics/sedatives/hypnotics	5843 (35.4%)	1992 (12.1%)	3.99 (3.77, 4.23)	**<.001**
Mood stabilizers	409 (2.5%)	215 (1.3%)	1.93 (1.63, 2.27)	**<.001**
Stimulants	272 (1.6%)	204 (1.2%)	1.34 (1.12, 1.61)	**.002**

Significant OR and *P*-values in bold.

## Discussion

To our knowledge, this is one of the largest multi-institutional retrospective cohort studies on the association between early onset IBD Disease Activity and psychiatric disease burden as well as psychotropic medication use. In our cohort of 33 844 IBD patients, we found that newly diagnosed patients with active IBD within 6 months to 1 year of IBD diagnosis were consistently associated with significantly higher odds of psychiatric diagnoses and psychotropic medication prescriptions.

Prior studies have shown higher rates of anxiety, depression, substance use disorders, posttraumatic stress disorder, antidepressant use, and outpatient care utilization amongst patients with an IBD flare presenting to an emergency department.^5^ Other studies have shown similarly increased rates of anxiety and depression in IBD patients, especially if the disease is active.^6^ Our study provides novel clinical data from a large database providing an in-depth assessment of the impact of early onset disease activity on comorbid psychiatric disease burden. Moreover, it provides data on the risk of developing other psychiatric diagnoses along with psychotropic medication usage, which is much less described in the literature.

Optimal screening for mental health disorders is of paramount importance, even more so in patients with IBD. Due to the high prevalence of psychiatric disease in IBD and its impact on disease activity, several studies have recommended routine screening for mental health disorders, especially in the IBD cohort.^7,8^ It is important to apply appropriate interventions to at-risk individuals promptly. Given the findings of our study, it is imperative to start routine screening for psychiatric comorbidities early in the IBD course, with shorter intervals of screening for those with increased disease activity.

In our study, we also conducted a separate sensitivity analysis to compare outcomes between patients with active CD and nonactive IBD and patients with active UC and nonactive IBD. We compared them to the nonactive IBD cohort, as previous studies have shown a similar prevalence of psychiatric diagnoses between CD and UC patients.^9,10^ We were able to find similar trends of higher odds of psychiatric diagnoses and psychotropic medication prescriptions, except for bipolar disease diagnosis in UC patients, where the odds were similar between the active UC and nonactive IBD groups. Compared to their nonactive disease counterparts, patients with active CD and active UC had almost comparable increased odds of psychiatric disease diagnoses, except for increased odds for opiate use disorder (5.31 in CD vs. 3.15 in UC) and OCD (2.83 in CD vs. 1.73 in OCD) in the active CD cohort. The OR were comparable between the 2 disease groups for psychotropic medication usage. These findings highlight that psychiatric disease morbidity and psychotropic medication usage are prevalent in both subgroups of IBD patients and follow a similar pattern.

Our study has several strengths. It includes a large sample size thereby lending increased validity to our findings. The data is sourced from healthcare organizations from several regions of the United States to account for any geographic variability. We controlled for confounding factors by rigorous propensity score matching of several variables included in the Charleston comorbidity index (CCI) since chronic disease processes included in CCI are linked to psychiatric comorbidity and may impact studied outcomes. We also used both steroid usage (using multiple steroid medications) and elevated FC to define active IBD. This was done to provide a more specific definition for active disease and minimize false positives. Additionally, our rationale for using a high FC level [≥200 µg/g] was due to the fact this value has a higher specificity for disease activity compared to the FC cutoff of 100 (82% vs. 66%, respectively), thereby increasing specificity and minimizing false positives.^11^

Our study’s limitations include the use of billing codes, retrospective cohort analysis, and the inability to detect psychiatric comorbidities if patients received care outside of HCOs covered by TriNetX, which may have led us to underestimate the burden of psychiatric comorbidity. The former limitation is mitigated by including IBD-specific medication prescription as a factor in defining IBD, thereby increasing the sensitivity. Dawwas et al. have demonstrated that using the ICD-10 code for IBD diagnosis along with IBD-specific mediation prescription is associated with a higher positive predictive value for IBD diagnosis.^4^ Additionally, we are inferring from the database that the first use of an ICD-10 code for IBD likely corresponds to the first diagnosis, which might not always be true. We could not include new biologic initiation as another adjunct to define active IBD, as this criterion was used to define new IBD diagnoses in our cohort. Moreover, due to the limitations of the database, endoscopic evaluation could not be used to highlight a disease flare.

Another limitation owing to the study design is that steroid usage (used to define active IBD) has been associated with the worsening of underlying mental health symptoms, which could, in theory, confound the results. However, most of the psychiatric adverse effects associated with steroid use described in the literature are limited to psychotic, mood, and cognitive effects occurring within a couple of weeks of steroid initiation and subside with stopping steroid medications.^12^ In our study, we looked at additional psychiatric diagnoses and looked at psychiatric disease outcomes occurring beyond the acute period of steroid initiation, thereby limiting the effect of this potential confounder. Moreover, we conducted a separate sensitivity analysis to look at active IBD defined solely by elevated FC to mitigate this potential bias. We were able to show that these IBD patients also had higher odds of a majority of psychiatric diagnoses and psychotropic medication usage. For the outcomes where we did not achieve statistical significance, it was mainly due to a lower number of subjects leading to less power to detect a significant difference.

In conclusion, active IBD shortly after the IBD diagnosis is associated with a higher incidence of psychiatric diagnoses and psychotropic medication usage. This holds for CD and UC patients separately as well. This underscores the importance of behavioral and psychosocial health in IBD and calls for early and routine assessment of comorbid psychiatric disease in patients with active IBD. Further studies are needed to understand the impact of IBD disease activity on comorbid psychiatric disease, optimal screening strategies, and prognosis.

## Supplementary Material

otae066_suppl_Supplementary_Material

## Data Availability

Additional data available in Supplementary Material.
