# Supplementary material for: Early Onset Active Inflammatory Bowel Disease Is Associated With Psychiatric Comorbidities: A Multi-Network Propensity-Matched Cohort Study
Source: Crohns Colitis 360. 2024 Dec 20;7(1):otae066. doi: 10.1093/crocol/otae066 (PMC11694680; doi:10.1093/crocol/otae066)
Supplement: otae066_suppl_Supplementary_Material [file otae066_suppl_supplementary_material.docx]

**Supplement 1 A, B**

A. Psychiatric disease diagnoses with their respective ICD-10 codes listed below:

| **Psychiatric Diagnoses** | **ICD-10 Code** |
| --- | --- |
| Depressive Disorder | F32-F33 |
| Anxiety Disorder | F41 |
| Bipolar Disorder | F31 |
| Alcohol Use Disorder | F10 |
| Opiate Use Disorder | F11 |
| ADHD | F90 |
| OCD | F42 |

B. Complete list of all specific medications under each class of psychotropic medication (based on RxNorm codes):

**Antidepressant:**

704 amitriptyline 722 amoxapine 42347 bupropion 2556 citalopram 3247 desipramine 734064 desvenlafaxine 3638 doxepin 72625 duloxetine 321988 escitalopram 2119365 esketamine 4493 fluoxetine 5691 imipramine 6130 ketamine 1433212 levomilnacipran 15996 mirtazapine 31565 nefazodone 7531 nortriptyline 36437 sertraline 32937 paroxetine 8886 protriptyline 10737 trazodone 10834 trimipramine 39786 venlafaxine 1086769 vilazodone 1455099 vortioxetine 6011 isocarboxazid 8123 phenelzine 9639 selegiline 10734 tranylcypromine

**Antipsychotics:**

784649 asenapine 89013 aripiprazole 1658314 brexpiprazole 1667655 cariprazine 2403 chlorpromazine 2626 clozapine 4496 fluphenazine 5093 haloperidol 73178 iloperidone 6475 loxapine 2275602 lumateperone 1040028 lurasidone 61381 olanzapine 679314 paliperidone 8076 perphenazine 8331 pimozide 51272 quetiapine 35636 risperidone 10502 thioridazine 10800 trifluoperazine 115698 ziprasidone

**Substance use treatments:**

82819 acamprosate 1819 buprenorphine 3554 disulfiram 7242 naloxone 7243 naltrexone 38404 topiramate

**Anxiolytics/Sedatives/Hypnotics:**

596 alprazolam 1827 buspirone 2356 chlordiazepoxide 2598 clonazepam 2353 clorazepate 3322 diazepam 461016 eszopiclone 4501 flurazepam 5553 hydroxyzine 6470 lorazepam 6960 midazolam 7781 oxazepam 8004 pentobarbital 596205 ramelteon 1547099 suvorexant 10355 temazepam 10767 triazolam 74667 zaleplon 39993 zolpidem

**Mood stabilizers:**

2002 carbamazepine 6448 lithium 28439 lamotrigine 32624 oxcarbazepine 38404 topiramate

**Stimulants:**

725 amphetamine 3288 dextroamphetamine 352372 dexmethylphenidate 700810 lisdexamfetamine 6816 methamphetamine 6901 methylphenidate
